# An e-learning pediatric cardiology curriculum for Pediatric Postgraduate trainees in Rwanda: implementation and evaluation

**DOI:** 10.1186/s12909-022-03222-z

**Published:** 2022-03-16

**Authors:** Emmanuel Rusingiza, Faraz Alizadeh, Traci Wolbrink, Barbra Mutamba, Samuel Vinci, Elizabeth L. Profita, Steven Rulisa, Lisa DelSignore, Jessica Solis, Robert Geggel, Kim Wilson

**Affiliations:** 1 University Teaching Hospital of Kigali, Kigali, Rwanda; 2 School of Medicine and Pharmacy, University of Rwanda, Kigali, Rwanda; 3 Boston Children’s Hospital, Boston, MA USA; 4 Harvard Medical School, Boston, MA USA; 5 University of Washington School of Public Health, Seattle, WA USA; 6 Lucile Packard Children’s Hospital Stanford, Palo Alto, CA USA; 7 Stanford University School of Medicine, Palo Alto, CA USA; 8 Tufts Children’s Hospital, Boston, MA USA; 9 Tufts University School of Medicine, Boston, MA USA

**Keywords:** E-learning, Pediatric cardiology, Resident training, Rwanda, Flipped classroom

## Abstract

**Background:**

Access to pediatric sub-specialty training is a critical unmet need in many resource-limited settings. In Rwanda, only two pediatric cardiologists are responsible for the country’s clinical care of a population of 12 million, along with the medical education of all pediatric trainees. To strengthen physician training opportunities, we developed an e-learning curriculum in pediatric cardiology. This curriculum aimed to “flip the classroom”, allowing residents to learn key pediatric cardiology concepts digitally before an in-person session with the specialist, thus efficiently utilizing the specialist for additional case based and bedside teaching.

**Methods:**

We surveyed Rwandan and US faculty and residents using a modified Delphi approach to identify key topics in pediatric cardiology. Lead authors from Rwanda and the USA collaborated with OPENPediatrics™, a free digital knowledge-sharing platform, to produce ten core topics presented in structured videos spanning 4.5 h. A mixed methods evaluation was completed with Rwandan pediatric residents, including surveys assessing knowledge, utilization, and satisfaction. Qualitative analysis of structured interviews was conducted using NVivo.

**Results:**

Among the 43 residents who participated in the OPENPediatrics™ cardiology curriculum, 33 (77%) completed the curriculum assessment. Residents reported using the curriculum for a median of 8 h. Thirty-eight (88%) reported viewing the curriculum on their personal or hospital computer via pre-downloaded materials on a USB flash drive, with another seven (16%) reporting viewing it online. Twenty-seven residents viewed the course during core lecture time (63%). Commonly reported barriers to utilization included lack of time (70%), access to internet (40%) and language (24%). Scores on knowledge assessment improved from 66.2% to 76.7% upon completion of the curriculum (*p* < 0.001) across all levels of training, with most significant improvement in scores for PGY-1 and PGY-2 residents. Residents reported high satisfaction with the visuals, engaging presentation, and organization of the curriculum. Residents opined the need for expanded training material in cardiac electrocardiogram and echocardiogram and requested for slower narration by foreign presenters.

**Conclusion:**

Video-based e-learning via OPENPediatrics™ in a resource-limited setting was effective in improving resident’s knowledge in pediatric cardiology with high levels of utilization and satisfaction. Expanding access to digital curriculums for other pediatric sub-specialties may be both an effective and efficient strategy for improving training in settings with limited access to subspecialist faculty.

**Supplementary Information:**

The online version contains supplementary material available at 10.1186/s12909-022-03222-z.

## Background

Pediatric non-communicable diseases contribute significantly to the global burden of disease, yet many low resource settings lack access to pediatricians and pediatric subspecialists, limiting patient care and medical training [1, 2]. In Rwanda, the Human Resources for Health program aims to build capacity for high quality clinical care utilizing a collaboration between the Rwandan Ministry of Health and a consortium of US academic partners to support expanded residency programs in pediatrics and other specialties, with faculty from the United States rotating to Rwanda for three to 12 month teaching positions [3].

Despite these collaborative efforts, access to pediatric subspecialty care training remains a critical unmet need, with additional training in pediatric cardiology identified internally as one of the priority areas [4]. While the exact numbers of children with cardiac disease in Rwanda are not available, estimates suggest that approximately 41,000 of the country's 410,000 live births (1 in 100) are born with congenital heart disease (CHD) with an even greater number impacted by acquired rheumatic heart disease (RHD) [5–8]. Currently, the country’s pediatric cardiology needs are served by two pediatric cardiologists, or one pediatric cardiologist per 2.7 million children, in contrast with the one per 25,000 children in the United States [9, 10]. These two pediatric cardiologists assume the responsibility for both clinical care and training other physicians in core cardiac skills.

E-learning, a broad term used to describe electronically mediated learning, offers one promising model of building capacity and training clinicians in areas with limited human resources and clinical skills [11–13]. E-learning offers significant potential for scale-up of medical training in limited resource settings with few subspecialty educators. As such, increasing access to e-learning opportunities for healthcare professionals has been a priority for the Rwandan Ministry of Health. Clinician-educators from Rwanda’s University Teaching Hospital in Kigali (CHUK) partnered with collaborators at Boston Children’s Hospital to develop an e-learning curriculum in pediatric cardiology for Rwandan residents through OPENPediatrics™ (www.openpediatrics.org), a web-based learning system whose mission is to make educational programs universally accessible to clinicians and medical trainees across the globe [14, 15]. Studies have shown positive results in learner engagement and knowledge gain through OPENPediatrics™ using their engaging learning modules with pre- and post-video assessments, instruction by video narration with integrated knowledge checks, summary documents with high-yield learning points, and interactive online simulators.

Given that specialized training in pediatric cardiology includes the understanding and appreciation of complex anatomy and physiology for patients with CHD and RHD, the partnership with OPENPediatrics™ would offer the technology to effectively present these topics to learners in a clear and interactive visual format.

The purpose of this study was to assess learner’s knowledge gain about key pediatric cardiology topics utilizing an e-learning curriculum and a flipped classroom approach where Rwandan pediatric residents watched videos and completed associated pre- and post-test assessments online prior to engaging in hands-on bedside training with local subspecialists. Secondary aims included evaluation of the feasibility and user experience of this curriculum in the resource limited setting. We hypothesized that the use of e-learning in a flipped classroom model would improve pediatric residents’ knowledge and comprehension of key pediatric cardiology topics, in addition to being feasible and well-liked by learners in our limited resource setting.

## Methods

### Curriculum development

To define the most relevant topics in pediatric cardiology in Rwanda, we reviewed the competencies in Pediatric Cardiology of the University of Rwanda Residency Program and the Accreditation Council for Graduate Medical Education in the United States. The Rwandan pediatric residency competencies were developed by pediatric staff in collaboration with partners and approved by the School of Medicine, the College of Medicine and Health Sciences, the Academic Senate and finally the Rwanda High Education Council. We recruited pediatric faculty at the University of Rwanda and pediatric cardiologists with global health experience from Boston Children’s Hospital to participate in a survey to identify core curriculum topics using a modified Delphi approach. Following the first round of ranking 23 topics comprised of cardiac diagnostic skills and pediatric cardiac diagnoses, 12 faculty participants participated in a second Delphi ranking round. Following this comprehensive review, we devised a proposed curriculum with ten core topics that the authors agreed that all pediatricians in Rwanda should have competency in.

We recruited lead authors from the pediatric cardiology faculty at the CHUK and Boston Children’s Hospital to create this curriculum, in collaboration with general pediatricians with global health experience in developing learning materials for low resource settings, as well as pediatric clinician-educators from OPENPediatrics™ who have expertise in the curation and best practices for delivery of online educational materials.

Five learning modules were created utilizing the ten topics. Each learning module included two to five lessons per module (additional file 1: Appendix 1). A lesson was defined as a single topic and consisted of an interactive video associated with content-related pre- and post-test questions in multiple choice format. For each of the ten topics, learning objectives and a storyboard were created by the lead authors, video instruction with engaging visual reinforcements was recorded by the OPENPediatrics™ production team, the content and language were reviewed by general pediatric global health faculty as well as by lead investigators to ensure appropriateness for the learners. Finally each video was peer reviewed by a pediatric cardiologist. Pre- and post-test multiple choice evaluations were developed and peer-reviewed for each topic.

### Implementation

The OPENPediatrics™ Pediatric Cardiology Curriculum was trialed in the University of Rwanda Pediatric Postgraduate Residency Training Program between August 2016 and January 2017. Residents were given access to the curriculum via multiple modalities including offline access via a USB flash-drive and online access with available downloading feature to view offline on learner’s personal electronic devices. Participants could watch the modules in the order of their preference. Additionally, each digital lesson was available for classroom viewings during four scheduled educational conference sessions that consisted of a pediatric cardiology faculty member providing further clarification, answering questions from residents and providing relevant clinical teaching at the bedside on the pediatric ward. Utilizing a flipped classroom approach, following completion of the e-learning module, learners participated in an in-person interactive case discussion lead by Rwandan pediatric faculty to consolidate and apply the information gained.

The curriculum remains available to the general public from any country online via the OPENPediatrics™ online open access platform [16], under the “Introduction to Cardiac Disease” course and can be accessed by any device connected to the internet in addition to being available for download for offline viewing. Ongoing IT support will be provided to Rwandan residents via the Rwandan Telemedicine Committee. The course objectives are appropriate for all pediatric medical trainees.

### Evaluation

Evaluation of the impact, satisfaction, utilization, and barriers of the OPENPediatrics™ Pediatric Cardiology Curriculum were evaluated using a mixed methods approach. Impact on knowledge gained was evaluated with pre- and post-test multiple choice assessments. Qualitative and quantitative assessment of the curriculum was completed by online survey and structured interviews.

### Quantitative evaluation

In April 2017, prior to accessing the e-learning curriculum, residents in the University of Rwanda Pediatric Postgraduate Residency Training Program completed an online survey assessing perceptions of e-learning as well as a 50-question pre-test knowledge assessment. Two months later, following completion of the OPENPediatrics™ Cardiology Curriculum and the four in-person review sessions, residents were invited to complete both a post-test knowledge assessment and a survey on self-reported competency, utilization, and satisfaction with the use of the digital training material. Survey questions used a five-point Likert response scale to assess learner perspectives. Survey responses were obtained using an online survey and downloaded as de-identified spreadsheet. Pre- and post-tests results were anonymized using unique study IDs that could not be linked to participation in the in-person review sessions.

### Quantitative data analysis, power calculation and sample size determination

Changes between pre- and post-test scores in knowledge were analyzed individually and collectively using paired sample t-tests and F-test, respectively. Wilcoxon sign rank test was used to analyze changes in learner response to survey questions utilizing five-point Likert scale. Learner responses to questions on utilization and satisfaction were analyzed using descriptive statistics, reflected as percentages.

Power was estimated for a paired sample t-test using a medium effect size of 0.5 standard deviation based on Cohen, with an alpha level of 5% for a two-tailed test. For power levels equal to 80%, the required sample size is 34 participants having complete data. Out of the 50 trainees in the residency program, we chose to enroll 45 participants in this study, anticipating a less than 100% survey completion compliance rate.

### Qualitative evaluation

Qualitative evaluation included both free response questions in the post-curriculum survey and post-curriculum in-depth interviews (IDIs). We conducted 12 IDIs of pediatric residents who were rotating through CHUK during a two-week qualitative data collection period and had completed the curriculum to explore their satisfaction and gain insight into their perceptions of strengths, weaknesses, and barriers associated with this specific pediatric cardiology e-learning curriculum as well as e-learning in general. IDIs took place in March of 2017 at CHUK. IDIs were led by author SV. The interviews were conducted on site within a private designated space as to encourage openness and reduce reporting bias. For all IDIs, only the participant and interviewer were present.

All IDIs utilized a semi-structured guide, which asked initial questions and provided probe questions if needed. The interviews were conducted in English, as all pediatric residents must be proficient in English as a requirement for their training. With permission from the participants, the interviews were audio-recorded for reflection and analysis by the research team. Full anonymous transcripts for each recording which were uploaded into NVivo software version 11 [17]. Transcripts were numbered, coded and sorted randomly, allowing for double coding of each transcript. Key themes were identified by each coder and discrepancies were resolved during review. Using a deductive approach, existing themes of digital learning in resource-limited settings were explored and emerging themes were identified using thematic analysis.

### Research ethics

Ethics approval for this study was obtained from University of Rwanda and Boston Children’s Hospital. Informed consent for participation in the evaluation of the OPENPediatrics™ curriculum was obtained at the initiation of the online survey. Study participants were assigned a study ID number for matching pre- and post-test response and data was collected anonymously for the sole purpose of evaluating the effectiveness of OPENPediatrics™. Aggregate data without unique identifiers was stored digitally and aggregate results were shared with University of Rwanda pediatric faculty.

## Results

Forty-five pediatric residents were initially enrolled in the study but two left the residency program in the middle of the study. Forty-three residents completed the post-curriculum survey. Thirty-three pediatric residents (77%) completed both pre and post-test assessments. Twenty-seven (63%) viewed at least part of the course during one or more of the four in person educational conferences which were followed by a question-and-answer review session with the local pediatric cardiologist.

### Pre- and post-test results

**Fig. 1:**
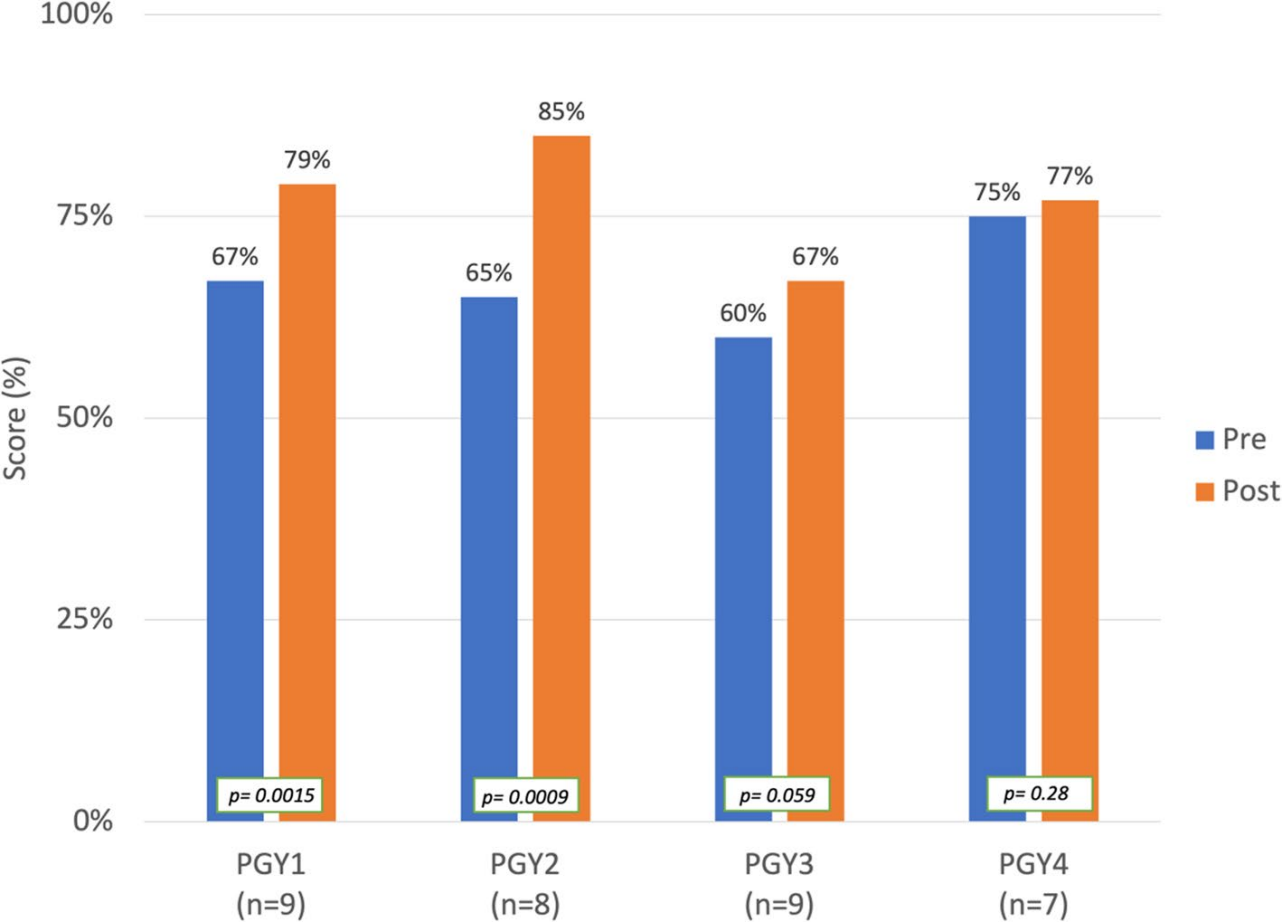
Mean Percent Correct Test Scores in Pre-test and Post-Test by Post Graduate Year (PGY) Level

Mean test scores of all thirty-three pediatric residents on knowledge assessment improved from 66% on the pre-test to 76% on post-test. The greatest improvement in test scores were seen in first year (*n* = 9; 67% to 79%; *p* = 0.015) and second year residents (*n* = 8; 65% to 85%; *p* < 0.01). Full results shown in Fig. 1. F test revealed improvement in the change in test scores between pre- and post-tests when residents of all training levels were included (*p* = 0.03).

### Quantitative survey results

Twenty-seven (63%) learners viewed all five modules, with 14 (32%) viewing three or four modules, and three (7%) viewing just one or two modules. The congenital heart disease module was the most viewed module with 40 (93%) residents completing it. Thirty-eight (88%) completed the rheumatic heart disease module, 37 (86%) completed the heart failure module, 35 (81%) completed the cardiac assessment module, and 33 (78%) completed the cardiac anatomy and physiology modules. Wilcoxon sign rank test revealed statistically significant improvement in self-reported confidence in all five categories (*p* < 0.001 for each module).

The median time to complete modules was eight hours (interquartile range (IQR): 5–10 h). Thirty-eight (88%) learners viewed the curriculum on their personal or hospital computer via a USB flash drive while seven (16%) viewed it online and 24 (55%) used the curriculum in reference to a specific patient (n.b. these options were not mutually exclusive).

**Fig. 2:**
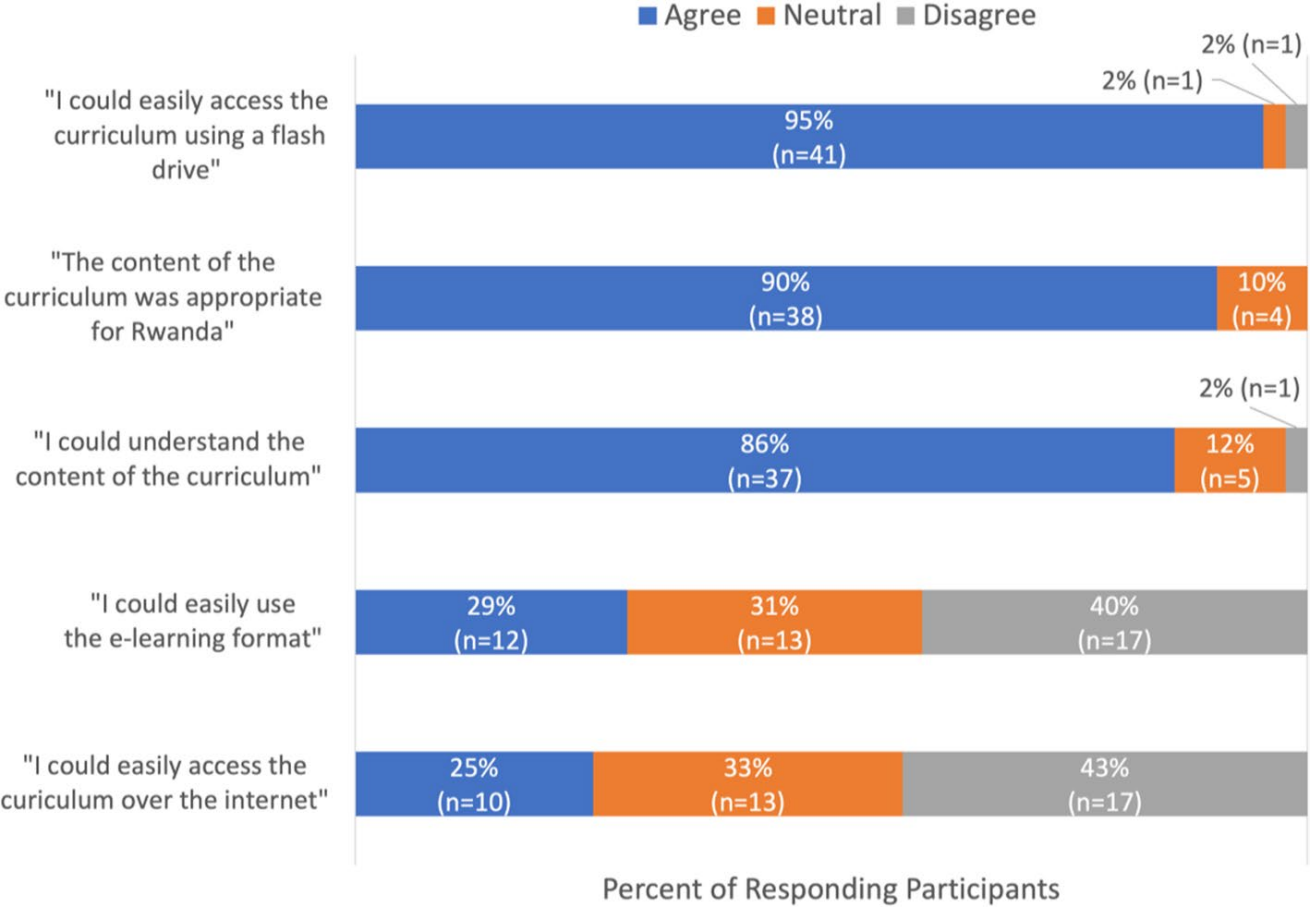
Participant Feedback on the Content of and Access to the Curriculum

Figure 2 demonstrates resident perception of the content of and access to the curriculum, with largely positive feedback on curriculum content and ability to access the content on a computer through the USB flash drive, but more varied response in participants ability to learn from e-learning format and ability to access the material using the internet.

Each question was not mandatory to answer and therefore has a different number of total respondents (*n* = 40, 42, 43, 42, 43, respectively).

**Fig. 3:**
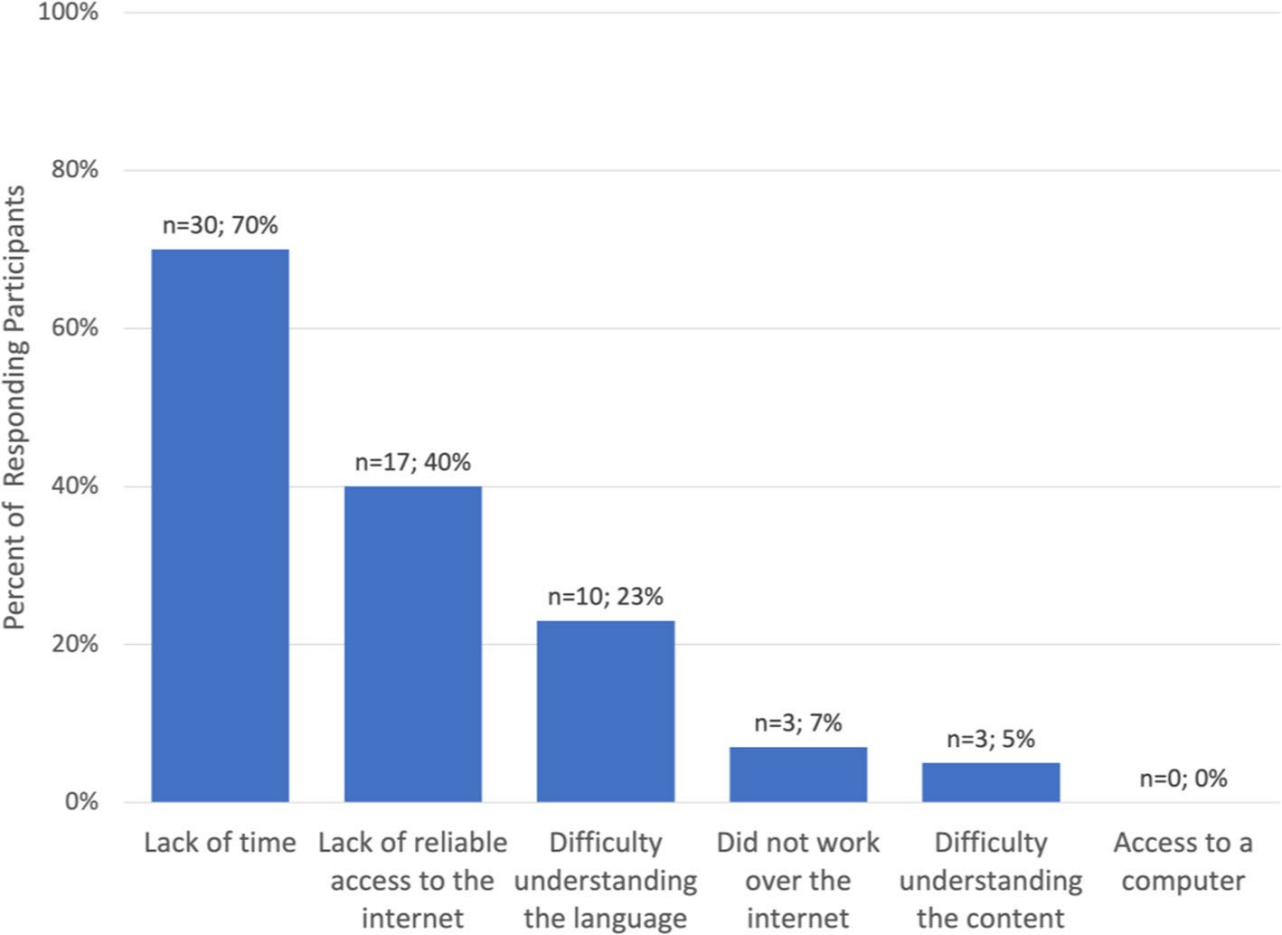
Self-Reported Barriers to Learning from this Online Curriculum

Figure 3 reveals participant self-reported feedback on major barriers which limited their ability to learn from the e-learning curriculum, with 70% reporting lack of time, 40% with lack of reliable access to the internet, and 23% with difficulty understanding the English instruction.

### Qualitative survey and interview results

Thirty residents responded to the post-curriculum survey. Resident feedback included suggestions for presenters to speak more slowly (six residents), to include courses on interpreting electrocardiograms (ECG) and echocardiograms (five residents), and to repeat this course every year (three residents).

Twelve residents participated in IDIs. Common themes are listed in Table 1. Learners enjoyed that the videos were short and concise, giving them an opportunity to review material as needed at their own pace. One resident described the e-learning modules as particularly helpful because they were able to watch the specific video right after seeing a patient with the associated condition:

“I remember when I was at Butare [Hospital], I had a chance to watch and it was very interesting because I was following two kids with rheumatic heart disease. When I saw the patients, I went home and watched the videos on rheumatic heart disease and everything was very clear for me. I wish to have every topic in that format of videos so when you have a patient you go home and search for video on that specific condition and can review so that you can better understand. I find it very helpful.”

**Table 1:** Common themes from in-depth interviews of Rwandan Pediatric Post-Graduate Trainees after completion of the Open Pediatrics^TM^ pediatric cardiology curriculum

Themes
Visual aids assist learners in understanding cardiac topics and make modules more engaging
Short and concise videos give learners the opportunity to review material at their own pace, before or after caring for a patient with a particular condition
It is helpful to provide educational multimedia by USB flash disk rather than via the internet given unstable internet conditions
Presenters should speak more slowly when targeting a population of learners with English as a second language. Learners found the use of subtitles helpful in this context
Learners do not yet feel comfortable interpreting electrocardiograms on their own and request more assistance with this topic
Residents have busy clinical schedules and do not have enough dedicated study time to participate freely in online learning
A “flipped classroom model” was beneficial to participants, allowing residents to consolidate their learning and ask more advanced questions when in person with the content expert

One common theme opined in both the survey and interviews was that residents had difficulty utilizing these video resources due to time constraints within their busy clinical schedules. One resident nicely summarized the problem by saying:

The issue is just finding a good time to watch them. It is almost impossible to watch them at work. You have to watch them after work, or when you are off, or on the weekend if you are traveling. Instead of watching a movie or listening to music you can watch the videos on your tablet.

Some residents, clinical schedule permitting, were able to watch the videos as part of a flipped classroom model having an in-person session with the local cardiologists following the video. All participating residents said this blend of in person and virtual learning was beneficial as it combined the benefits of both, allowing residents to ask more advanced questions once they understood the basics. One resident stated:

It is very important for us [to have an in-person session following the video] because if you have a challenge or there is something that you don’t understand, [the instructor] is there to explain to you, give you examples, help you understand very well those topics. It is very important, especially in cardiology, it was very helpful to have him with us.

## Discussion

E-learning offers a potential solution for the challenges of educating medical trainees in settings with workforce shortage of pediatric subspecialists. This study analyzed the efficacy of a video-based pediatric cardiology curriculum for pediatric residents in Rwanda, hosted on OpenPediatrics™ online platform and offered on USB flash drives. Most residents improved scores in knowledge assessment from pre-test to post-test, with greatest improvement in the more novice residents. Qualitative feedback was largely positive, with notable strengths of the curriculum including that the material was relevant to their clinical care, the presentation was concise and engaging, and that the e-learning format allowed participants to review material at their own pace. Participants reported that some barriers to learning including a lack of time, lack of reliable access to the internet and difficulty understanding the English instructions, particularly when the narrators spoke quickly.

E-learning and flipped classroom models have been shown to be an effective educational model in low- and middle-income countries (LMICs). In a systematic review of e-learning in low resourced settings, most published articles used a blended learning model, or “flipped classroom”, where learners study core concepts on electronic formats on their own before attending an in-person session where they can consolidate their learning, ask follow-up questions and practice what they’ve learned [13]. In our study, a flipped classroom model was offered to residents based on their availability and clinical assignments. Residents were invited to an in-person review with the local pediatric cardiologist. They could either watch the course at home before the session or in person immediately before the review session. Residents who attended these sessions found them very helpful, allowing them to ask questions and clarify topics they did not understand initially. Furthermore, e-learning is an innovative way for international academic institutions to partner with one-another to facilitate training in areas of workforce shortages. Numerous international studies have shown that these partnerships have led to successful e-learning models, combining the medical and technological expertise of both partner sites [18, 19]. Our curriculum is another successful example of a partnership combining the local pediatric cardiology expertise at the CHUK, with the experience of a large heart center at Boston Children’s Hospital, and the technological expertise, videography skills, and online platform of OpenPediatrics™.

One of the successful characteristics of this pediatric cardiology e-learning curriculum relates to the use of best practices of adult learning theory [20] and multimedia learning theory [21], the latter of which focus on reducing cognitive overload. As opposed to traditional classrooms at the primary, secondary and undergraduate level, adult learning requires succinct delivery of information and regular repetition [22]. Multiple learners noted that the short videos in this curriculum were more engaging compared to more traditional one-hour long lectures. Furthermore, adult learners need to understand why the material is important to learn and how the material is relevant to their daily lives, best exemplified by the resident who noted in his IDI that the videos on RHD were most useful after seeing two patients with RHD earlier that day.

Many educational programs in LMICs have reported that reliable access to internet remains a major barrier to e-learning [23–25]. While participants in Rwanda reported that all trainees have access to a computer and mobile phone, internet at the hospital is sometimes unreliable and purchasing data bundles through a mobile phone provider can be costly. Pre-downloaded videos offer a good solution to this challenge; however, some e-learning features require an internet connection (e.g. discussion boards, some interactive features, online quizzes, etc.). Furthermore, many LMICs have reported that tailoring the educational material to the local language and cultural context remains to be a barrier [18, 26, 27]. Learners in our study did report that they found the speed of English narration to be difficult to understand. Of note, Rwanda is in a period of transition as the national language changed from French to English when it became part of the Commonwealth in 2008. As a result, there is a currently a transition where many of the older medical residents grew up studying in their primary and secondary schooling in French while the new medical residents are more familiar studying in English since primary and secondary school. All the residents who studied medicine in Rwanda received their training in English and English is the primary language of instruction at the University of Rwanda and CHUK currently. However, even for learners with fluency in English, e-learning program developers must take into consideration regional differences in accent and intonation. When available, it is preferable to have local instructors speaking in the preferred local language. However, for many curricula with an international audience without a single unified language and accent, such as OpenPediatrics™, this will not be possible, and instructors should be reminded to speak slowly and with clear articulation and subtitles should be added whenever possible.

Our study has numerous limitations. First, the context of medical training in Rwanda is unique and may not be generalizable to all post-graduate medical trainees in LMICs. Our assessment of knowledge was limited as we did not achieve our goal sample size to complete the pre- and post-tests and we did not have any long-term follow-up in knowledge assessment. Not all residents were able to attend the in-person review sessions and we were not able to analyze the pre- and post-test scores with their participation in the in-person review sessions, making it difficult to isolate the effects of e-learning component and the in-person review on improvement in scores. IDIs may be limited by conformity bias where residents may be influenced to speak positively about the content. While our curriculum development included input from Rwandan clinicians, due to the limited number of pediatric cardiologists in Rwanda, the majority of the modules were taught by US based clinicians. Finally, while our curriculum appears to improve the knowledge of trainees, we have not demonstrated knowledge retention nor improved clinical skills.

## Conclusion

E-learning holds significant promise as part of the solution to the global workforce shortage in pediatric subspecialities in low resource settings. A video-based e-learning pediatric cardiology curriculum hosted by OpenPediatrics™ showed promising results in both quantitative improvements in knowledge assessment and in qualitative learner experience. Major strengths include that the videos were concise, engaging, and convenient, while some barriers include lack of time and access to reliable internet that was mitigated by pre-downloaded videos. Future iterations should consider focus on content revision with increased ability to practice skills, such as interpret ECGs. In settings with limited access to pediatric sub-specialists, e-learning curriculums may be both an effective and efficient strategy for increasing access to high quality medical education.

## Supplementary Information

**Additional file 1.** 

## Data Availability

The datasets used and/or analyzed during the current study are available from the corresponding author on reasonable request.

## Abbreviations

CHD: Congenital heart disease
CHUK: University Teaching Hospital in Kigali
ECG: Electrocardiogram
IDIs: In-depth interviews
IQR: Interquartile range
RHD: Rheumatic heart disease
