# Supplementary material for: An e-learning pediatric cardiology curriculum for Pediatric Postgraduate trainees in Rwanda: implementation and evaluation
Source: BMC Med Educ. 2022 Mar 16;22:179. doi: 10.1186/s12909-022-03222-z (PMC8925059; doi:10.1186/s12909-022-03222-z)
Supplement: Supplementary file 1 — Additional file 1. [file 12909_2022_3222_MOESM1_ESM.pdf]

APPENDIX ONE:
Curriculum Overview

Module 1: Cardiology Assessment and Evaluation
Lesson 1: **Cardiac History & Exam**
Lesson 2: **Interpretation of Pediatric CXR**
Lesson 3: **Evaluation of CXR if Suspected Heart Disease**
Lesson 4: **Introduction to EKG**

Module 2: Cardiac Anatomy and Physiology
Lesson 1: **Basic Cardiac Anatomy and Physiology**
Lesson 2: **Fetal Circulation**

Module 3: Congenital Heart Disease
Lesson 1: **Cyanotic Congenital Cardiac Defects**
Lesson 2: **ASD and VSD**
Lesson 3: **Tetralogy of Fallot**
Lesson 4: **Murmurs**
Lesson 5: **Arrhythmias**

Module 4: Congestive Heart Failure
Lesson 1: **Pathophysiology & Diagnosis of Heart Failure**
Lesson 2: **Management of Congestive Heart Failure**

Module 5: Rheumatic Heart Disease
Lesson 1: **Acute Rheumatic Fever**
Lesson 2: **Rheumatic Heart Disease Additional Materials**
